# Police officers’ experiences of supportive and unsupportive social interactions following traumatic incidents

**DOI:** 10.3402/ejpt.v4i0.19696

**Published:** 2013-03-15

**Authors:** Rachel Evans, Nancy Pistrang, Jo Billings

**Affiliations:** 1 Research Department of Clinical, Educational and Health Psychology, University College London, London, UK; 2 Berkshire Traumatic Stress Service, Reading, UK

**Keywords:** Post-traumatic stress disorder, emergency services, qualitative interviews, social interactions, relationships, social constraints, partners, peers, coping

## Abstract

**Background:**

Police officers are routinely exposed to potentially traumatic incidents yet the majority do not develop post-traumatic stress disorder (PTSD). Social support has been identified as one factor that may maintain wellbeing in this population, although what constitutes supportive or unsupportive interactions is unclear.

**Objective:**

To explore police officers’ experiences of supportive and unsupportive interactions following distressing incidents.

**Method:**

Semi-structured interviews were conducted with 19 police officers. Transcripts were analysed using Braun and Clarke's (2006) thematic analysis approach.

**Results:**

Participants described a range of supportive interactions with colleagues, friends, and family, as well as social constraints that hindered interactions. Ambivalence about talking about the impact of distressing events was striking throughout the accounts. The context and source of available support, as well as beliefs about talking, influenced their interactions. Humour was a central feature of interactions with colleagues; more emotional talk occurred with partners and close family, albeit with officers limiting details in order to protect others.

**Conclusions:**

The findings provide tentative insights into the processes of social support that may contribute to the resilience of police officers following traumatic incidents. Further research is needed to examine whether the experiences of supportive and unsupportive interactions differ for those with and without PTSD.

Emergency service work, undertaken by police officers, fire fighters, and ambulance paramedics, carries an inherent risk of exposure to situations that many would find traumatic. Therefore, it is not surprising that this population has been considered psychologically “at risk” with higher lifetime prevalence rates of mental health difficulties such as post-traumatic stress disorder (PTSD) than the general population (Clohessy & Ehlers, 1999). However, despite exposure to numerous potentially traumatic events, the majority of emergency service personnel do not show signs of psychological distress and, in fact, some report positive effects of emergency work (Moran & Colless, 1995; Shakespeare-Finch, Smith, Gow, Embelton, & Baird, 2003). Research with this population is important for understanding the factors conferring risk or resilience to traumatic incidents, with implications for both practical support programs and theoretical accounts of PTSD in the clinical field.

Meta-analytic reviews of studies conducted across a range of populations indicate that a lack of social support is one of the strongest risk factors for PTSD after trauma exposure (Brewin, Andrews, & Valentine, 2000; Ozer, Best, Lipsey, & Weiss, 2003), a finding replicated in studies with emergency service personnel (Prati & Pietrantoni, 2010). However, less is known about the mechanisms underlying this relationship. Factors proposed to influence the relationship between social support and PTSD in the emergency service population include attitudes towards emotional expression (Hoyt et al., 2010; Lowery & Stokes, 2005), the source of support (e.g., work supervisors or friends; Stephens & Long, 1999), and the culture of teams and professionals (Bacharach & Bamberger, 2007). However, as a body of research, insufficient attention has been paid to the features of “supportive” or “unsupportive” interactions, thereby limiting our understanding of the processes underlying links between social support, PTSD, and other related variables. In part, this may be due to a reliance on quantitative measures of social support, which conceptualise it as something given and received, rather than a dynamic process (Guay, Billette, & Marchand, 2006). It may also reflect the individualistic focus taken by predominant theoretical accounts of PTSD (Brewin, Dalgleish, & Joseph, 1996; Ehlers & Clark, 2000; Foa, Steketee, & Rothbaum, 1989).

The social–cognitive processing model (Lepore, 2001) explicitly addresses the interplay between individual factors and interpersonal processes; it proposes that an individual's social environment can promote or deter willingness to talk about a traumatic event, in turn influencing the level of cognitive processing and adaptive adjustment. Unsupportive, unreceptive, and critical responses from others (e.g., “I've not got time for this”, “You've only got yourself to blame”) are thought to increase risk of PTSD by discouraging talking and increasing cognitive avoidance and suppression of the trauma-related material. To date, with few exceptions (e.g., Farnsworth & Sewell, 2011), this model has received little research attention in the context of emergency service work.

## Objectives

In reviewing the literature on social support and trauma, Guay et al. (2006) suggest that it is premature to conclude what is “supportive” and indicate a role for qualitative research to better understand the processes underlying this complex relationship. The present study used a qualitative approach to explore the nature of social support interactions from the perspective of police officers who had experienced distressing incidents but had not developed PTSD; the aim was to understand the types of support processes that might promote resilience. It addressed the following questions, informed in part by Lepore's (2001) social–cognitive processing model of adjustment to trauma:

1. What are police officers’ experiences of supportive and unsupportive interactions following potentially traumatic incidents?
2. Do interactions differ on the basis of the context and source of support (i.e., at work with colleagues and supervisors, or outside of work with family and friends)?
3. How do supportive/unsupportive interactions facilitate/hinder the processing of traumatic incidents?

## Method

### Procedure

Police officers in two urban areas of the UK (London and Birmingham) were recruited using a snowballing approach (Patton, 2002). Volunteers who expressed an interest were screened by telephone or email for two eligibility criteria: (1) a minimum of 2 years’ experience (to ensure they had completed their training and probation period and would have had exposure to “traumatic” incidents); and (2) no self-reported current or prior history of PTSD (ascertained by asking systematically about PTSD symptoms). A National Health Service ethics committee granted ethical approval; all individuals gave informed consent to take part.

### Participant characteristics

Nineteen police officers (13 men, six women) took part in the study; their mean age was 36 (range: 25–50). The majority (N = 16) described their ethnicity as “White British”, two as “White European”, and one as “Mixed Heritage”. All but four were married or living with a partner. Three husband and wife couples participated (i.e., three men and three women, each interviewed separately).

The median length of time in the police service was 7 years (range: 2.5–28 years). Ranks included police constable (N = 9), detective constable (N = 2), police sergeant (N = 5), and detective inspector (N = 3). Officers worked in first response teams, safer neighbourhood teams, and specialised departments such as murder investigation and road policing. All were employed full-time.

### Semi-structured interview

A semi-structured interview schedule was designed specifically for the study. A pilot interview highlighted the need to elaborate on the meaning of “traumatic” incidents as this was a term officers reserved for major incidents (e.g., 7/7 bombing). The interview questions focused on the experience of events that participants identified as “traumatic” or “difficult”, i.e., ones that had “stuck with them” and caused them distress. The subsequent support received through interactions with others at work and outside work was explored in detail; both informal and formal support were considered, as these can be conceptualised as falling on a continuum of social support (Barker & Pistrang, 2002). Questions to elicit contextual information were asked at the start of the interview (e.g., about training for current policing role) and the end (e.g., advice to a “new recruit” on how to manage the impact of events). Interviews lasted between one to two hours; they were audio-recorded and transcribed verbatim.

### Qualitative data analysis

A thematic analysis approach (Braun & Clarke, 2006) was used. This is a systematic process of identifying patterns of meaning in participants’ accounts. The analysis comprised several steps: (1) familiarisation with the data through multiple readings of each transcript; (2) generating initial labels to capture the ideas expressed; (3) clustering labels representing similar ideas to produce a tentative list of themes for each interview; (4) comparing themes across interviews to create a “thematic map” of the data; and (5) defining and refining themes to produce a consolidated set of themes; these were grouped into three broad domains to provide an organising structure. Attention was paid to ensuring that each theme was supported by extracts from the interviews and “densely” described (Strauss & Corbin, 1998).

In accordance with good practice guidelines for qualitative research (Barker & Pistrang, 2005), “credibility checks” were undertaken throughout the analysis. The first author took the lead in the analysis; the second and third authors read a subset of transcripts and audited the first author's documentation of the analytic process. A consensus approach was used in order to avoid relying on a single researcher's interpretation of the data: the research team discussed different ways of conceptualising and representing the data, and modifications were made before reaching agreement on the final set of themes.

## Results

All participants identified distressing incidents which had “stuck with them” (Table 1). Incidents that caused most distress tended to be those that had personal relevance and heightened officers’ perceptions of their own or their loved ones’ vulnerability. Distress was described in a manner resembling PTSD symptoms (e.g., rumination, sensory memories) but below the clinical threshold in terms of level and functional impairment.

**Table 1:** Types of difficult incidents described in interviews

Type of incident	Key difficult feeling/thought	Relevant participant number
Dealing with distress of family members when informed of the loss of a loved one	Feeling more could have been done or unsatisfied with the outcome	P3, P4, P6
	Feeling the other's distress/putting oneself in the other's shoes (especially if perceived similarities between the victim and one's own situation)	P1, P2, P5, P11, P13, P14, P16, P19
Violent situation	Feeling targeted, out of control or vulnerable	P7, P15
Unusual death circumstances	Feeling uncomfortable, alone and ‘creepy’	P8, P17
Incidents involving ‘vulnerable’ people (e.g., children, elderly people or victims of domestic violence)	Perception of an innocent/vulnerable party, feeling the situation is unfair	P9, P10, P12
Major transport related incident	Thinking that they had let colleagues down by leaving the scene	P18

The analysis generated eight key themes organised into three domains (Table 2). The first domain refers to broad issues about whether to talk about incidents or not, pertaining to support both inside and outside work. However, it was clear that the nature of interactions differed depending on the source of support, and the second and third domains reflect this.

**Table 2:** Summary of themes from participants’ accounts

Domain	Themes	Subthemes
1. Dilemmas of talking	1.1 We don't need to talk	“You just get used to it”—hardened by exposure
		Talking about it isn't going to help
	1.2 Talking is risky	Emotion as a sign of weakness
		The importance of reputation
	1.3 Don't bottle up: “talk, talk, talk”	Talking helps
		But be careful who you talk to
2. The work context: informal interactions with colleagues and formal sources of support	2.1 Humour and banter	Helpful coping strategy
		Group process: saving face and gaining respect
		Sensitive use of humour—humour has its limitations
	2.2 “Dip in and out of chat”	Recognising signals of distress and requests to talk
		Selecting the person, time and place to talk
	2.3 Formal opportunities to talk	Ambivalence about formal services
		Importance of supervisors
3. Support outside work	3.1 A close relationship with someone who cares	Importance of partners
		“Selfless listening” and acceptance
	3.2 Protecting others	“Don't put that heartache on them”
		You need time off from work

### Domain 1: Dilemmas of talking

The most notable feature of the accounts was ambivalence towards talking about events that had a personal impact. Individual differences in general attitudes towards talking existed, but more striking were the mixed views within participant accounts.

#### Theme 1.1. We don't need to talk

Most participants indicated that they tended not to talk about difficult events because they were “used to” them and preferred to “get on with the job”, perceiving reflection on events and talking as unnecessary activities. They described “hardening” to the effects of witnessing traumatic incidents; this was seen as a natural effect of continuous exposure to incidents which altered officers’ perception of “normality”. Several officers described deliberately adopting strategies to “harden” or distance themselves from the effects of potentially distressing incidents.

It's that armour and I don't know how else you would deal with it, you've got to … it sounds so callous but you've so got to detach yourself … [Talking about an infant post mortem] You couldn't allow that to be a baby that you would nurture, that you would love because it would just break your heart. All it was, it was a dead body that you had to deal with … you just deal with it and you move on because otherwise you would crumble completely. [P10]

#### Theme 1.2. Talking is risky

Although all officers identified at least one situation which had personally affected them, there was a general reluctance to share this with others (several participants commented that they had talked more about events with the interviewer than they had with anyone else). Talking was described as a risky activity because it deviated from norms of British culture (keeping a “stiff upper lip”) and the “macho” culture of the police service, both of which emphasise “getting on with it”. Fears about appearing “weak” seemed to modify the way participants approached interactions. This was evident in the home context but was especially salient at work.

I think there's a real element of machismo and masculinity in the police force and it's a bit, sort of a faux pas to admit that things have really affected you … If I'd have come out and said ‘ah you know, that really affected me badly, let's go and sit down and have a cup of tea and talk about it’ I think you're straying into pink and fluffy territory there … saying ‘that made me feel sad’ is a bit too far. [P3]

The ability to remain calm and dispassionate in response to potentially emotive incidents was seen as the hallmark of a reliable police officer. All the officers feared sharing emotional reactions to events at work in case there was a detrimental effect on their reputation and career prospects.

There have been officers that are doing the shift that have shown that they can't deal with situations like that, and been very open about it—and they haven't got the respect from the shift, because the colleagues go ‘well, you're on your own if you're working with her, because she'd back away’ or whatever. So you don't want to be considered as one of those. [P7]

#### Theme 1.3. Don't bottle up: “talk, talk, talk”

Despite perceptions that talking was often unnecessary and risky, many participants strongly endorsed the view that talking helps. When asked in the interview what advice they would give to a new recruit, almost all participants warned against “bottling things up” by not talking about the impact of events.

The quickest piece of personal advice is for people to talk about it … Talk talk talk—get it out and speak to people! [P18]

Talking was depicted by many as a crucial activity for the maintenance of relationships (especially close relationships outside work) and talking with colleagues was described as a means by which officers realised they were “not alone”, which could lessen the shame or self criticism that might otherwise arise. The majority also saw talking as an outlet for emotion and a vital means of processing “traumatic” events.

… the more you talk about something, the more it becomes something you've told and your telling becomes part of the memory, as opposed to it being a really shiny, vivid thing inside your head—those images. [P17]

Having the option to talk was therefore highly valued. However, as a result of the perceived risks of emotional expression, participants spoke about needing to feel in control of the decision to talk. In both the work and non-work contexts, it was essential that the confidant was chosen carefully; they would need to be reliable, trustworthy and well known.

… don't be shy in coming forward but also, be careful about who you do speak to. [P9]

### Domain 2: The work context: informal interactions with colleagues and formal sources of support

Participants described ways in which they interacted with colleagues in an informal manner. Across all interviews, humour was emphasised as a central means for diffusing emotion and providing support in the aftermath of difficult events. However, informal “chat” with colleagues and formal opportunities to talk were also discussed. All forms of talk carried their own set of perceived risks and benefits.

#### Theme 2.1. Humour and banter

Humour was universally described as a helpful means of interacting with colleagues and talking about difficult events. The term “banter” was used interchangeably with humour and tended to denote interactions with the presence of more than two colleagues. Jokes about a comical aspect of an otherwise disturbing situation (e.g., dead bodies) were used as an “outlet” for awkward or uncomfortable emotions. Humour also acted to alter perceptions of an event, to change the emotional response at the time, and the memory of the situation in order to limit negative consequences.

[humour] makes the incident feel less serious, I suppose. (later in interview) … you, kind of, just diffuse from it, have a little joke about it and then you're on to the next one [incident]. Whereas we probably prolong it a little bit more, talking about it more seriously … [P7]

Officers highlighted the importance of using humour sensitively. Attention was paid to who was present; for example, humour would not be used in front of members of the public or other professionals who were not well known. Furthermore, the type of event and knowledge about the personal relevance to colleagues modified the timing and nature of humour.

I mean people wouldn't make jokes about a child death … [Also] I think if it's something that I refer to personally, like I put back to my own life, if somebody laughed at that I probably wouldn't find that very funny. [P11]

Humour was found to be a helpful way of indicating support to others in the team and banter was seen as crucial for team camaraderie. An officer's ability to banter had implications for their reputation, being seen as a sign of resilience and fitting in with the “macho” culture.

#### Theme 2.2. “Dip in and out of chat”

Participants described a process of approaching conversation about the impact of events and then withdrawing to avoid going “too deep” into emotional content. “Matter of fact talk” (rather than talk about emotions) and mixing humour into conversations acted to keep “chat” at a comfortable level.

… sometimes it will be serious and nine times out of ten it's jokes but it's a mixture, like a joke and then you'll say something and … then it will get a bit serious and awkward—one of you will realise what's going on and then you'll be back, crack a joke again … it's a way of getting it out. [P4]

As a result of concerns that showing emotion would risk reputation, officers described a complex, subtle system for picking up distress signals from colleagues.

You've got to know them as individuals, so is somebody acting out of character? Are they, for them, unusually quiet? Or, for them, unusually vocal? Because generally, it's not obvious, you won't see them as a crying, gibbering wreck in the corner … so signs for picking it up in general would be fairly subtle, and you have to be mindful of it. [P9]

These signals would be read by others who would respond with subtle indications of support (e.g., staying to have a cup of tea at the end of the shift or asking “what was the crack with that call out?”). Indirect opportunities to talk were generally preferred to directly stating a need to talk.

It needs to come out, generally outside of work or as a result of seeing something—people will take that opportunity to talk about things … when a sudden death call comes out, it's an instant trigger … it gives you an opportunity to say, ‘I went to this one once, it was so horrible’ … and that's acceptable and you can do that. [P17]

#### Theme 2.3. Formal opportunities to talk

Participants described a number of formal opportunities to talk, including group “debriefing” sessions, individual and group conversations with a “diffuser”, individual counselling, and individual trauma risk management sessions. Suspicion about the rationale for these services was voiced; some feared they were in place to detect and monitor “weak” officers.

The organisation is now very good at saying, we provide this type of welfare service for you to come … Are they doing it for the individual? If I'm honest, I don't think they are, I think they know, as an organisation it's expected of them and they're doing it to protect themselves, is my cynical view of it. [P5]

There was widespread concern that using formal sources of support (particularly counselling) could damage an officer's reputation and job prospects. Although they were reluctant to tell their colleagues, six participants had experienced at least one counselling session and were unanimous in the benefits of doing so.

It played on my mind but once I'd gone and spoke to the counsellor, spoken to someone at length about it, it just sort of cleared the air. And I felt, because I'd told somebody about it and they listened, afterwards I just kind of got used to it you know. [P13]

Mixed opinions were given on whether formal support services should be optional or mandatory. Some felt that being optional increased the stigma attached to service use; the following highlights this issue whilst also indicating an awareness of the discrepancy between officers’ public and private views:

We had counselling every six months … and everybody used to go ‘Oh I've got to see the counsellor this week’, but I tell you what … we all quite enjoyed it … I was so much calmer after speaking to her but it's something I'd never have done had I not been made to do it. [P12]

In comparison to formal support structures, participants emphasised the importance of supervisors as a source of support and influence on team attitudes towards talking. Increasing the level of supportive interactions with supervisors was seen as the change most likely to enhance officer wellbeing and perceptions of being supported at work. Supportive supervisors were described as “down to earth”, “approachable”, “doors always open” people, but about half of the officers also experienced unsupportive supervisors.

… he's [supervisor] quite an old fashioned sort of police officer, not the bloke you would sort of want to go in and have a chat with about a sudden death you'd just been to … If I went in and said ‘Governor, can I have a chat about the sudden death?’, he'd look at me as if I'd just asked to kill one of his children! [P3]

### Domain 3: Support outside work

In the majority of cases, support from a close other outside of work was highly valued. Most participants felt they would be more likely to speak to people at home, rather than at work, if they were personally affected by an event. However, perceived differences between those in and out of the police service led to concerns about being understood and a felt need to protect others, both of which moderated the way participants talked.

#### Theme 3.1. A close relationship with someone who cares

The majority of participants reported that they would want to talk in-depth to a loved one if they had been affected by an event at work and many described having the option to do so as vital.

I don't think talking about it to people at work is the release, the escape I need … it's speaking to people who I care about and who care for me and just having that comfort zone, that's what's important to me. [P16]

Officers in close relationships highlighted partners as a crucial source of support which often took the form of “talking things through” but also included gestures (e.g., cooking a favourite meal). A small subset of participants described avoiding speaking to partners for fear that they would “let go” and become emotional, something they personally felt uncomfortable with. If they did talk to a close other about a difficult incident, all emphasised the importance of the other person listening and being accepting of their reactions. This was described by some as “selfless listening”: it involved the other person putting aside questions or assumptions, listening and detecting what support was needed.

They'll just listen, they won't judge, they won't question, they'll just listen and go, ‘Are you alright?’ And that's what you need, isn't it? You just need that, that offload. [P8]

Seven of the participants were in relationships with another police officer; most viewed this positively because it helped them to feel freed up to talk frankly. However, it was common for these officers to describe limiting talk to avoid work “taking over”.

I find quite a lot of comfort in the fact that we're both in the job and I can talk to him in as much detail as I want … [but] we make a conscious effort not to talk about it too much. [P7]

#### Theme 3.2. Protecting others

Almost all participants described concerns about talking to non-police officers in case they said too much and upset the other person. Some thought it would be selfish to talk about their experiences because while it might unburden them, it would mean putting the burden on another. As a result of these concerns, participants described “vetting” the details of their accounts to avoid shocking or upsetting others.

… I never tell my wife that, I would never tell her that because I just think that would have really put the frighteners on her. [P15]

Many participants also avoided talking about work in the home context in order to “have a break” and protect themselves from burnout.

… because it's [the bad side of society] all we ever see when we're at work, you sometimes lose, like the light in your eyes, whereas when you go out with your friends or you see your family, they've still got that light because they haven't been blinded, they haven't been affected by it and that's quite refreshing, that's quite nice and I wouldn't want it to be any other way. [P6]

## Discussion

Police officers described a range of experiences of supportive interactions with colleagues, friends and family, as well as social constraints that hindered interactions. Although participants described rarely being affected by events at work, all had experienced some distressing events. Ambivalence about talking about the impact of such events was striking throughout the accounts. The context and source of available support, as well as beliefs about talking, influenced their interactions. Indirect banter and humour were central features of interactions with colleagues; more emotional talk occurred with partners and close family, albeit with officers limiting details in order to protect others.

Ambivalence about talking with colleagues and supervisors was linked to an implicit sense that emotions were “unspeakable” as officers “should cope”, an idea noted in another qualitative study of police officers (Howard, Tuffin, & Stephens, 2000). Emotional expression and seeking support were perceived as risky, potentially interfering with functioning effectively and damaging one's reputation. Such attitudes are likely to be shaped by the service culture (Kiely & Peek, 2002) as well as individual factors (e.g., alexithymia, McCaslin et al., 2006). Participants identified supervisors as a key influence on individual and team attitudes towards talking about difficult incidents.

Humour, which predominated in interactions with colleagues, appeared to have a positive function, providing distance from uncomfortable emotions and promoting the reappraisal of events as non-threatening. This is consistent with experimental findings that “good natured” humour diverts attention away from negative emotional processing and can evoke positive emotions that “undo” negative emotions (Samson & Gross, 2012). Humour was also described as a means of preserving masculine self-identity and a communication tool to broach difficult topics and implicitly acknowledge the emotionally difficult nature of events; both of these have been noted in other contexts, such as men talking about testicular cancer (Chapple & Ziebland, 2004). Furthermore, the social bonds forged through humour seemed to contribute to a sense of group cohesion and safety, which may have helped to modulate the emotional intensity of events the officers were exposed to (Charuvastra & Cloitre, 2008; Olff, 2012). Although other studies (e.g., Roth & Vivona, 2010; Wright, Powell, & Ridge, 2006) have noted the use of humour in this population, the accounts in the current study highlight its importance as a mechanism of social support and the subtlety with which it may be used.

Participants tended to seek opportunities to talk more seriously with people outside of work. In particular, partners were described as a key source of support, a finding common to the general population regarding help-seeking during times of stress (Barker, Pistrang, Shapiro, & Shaw, 1990). However, similar to other research with police officers (Freedman, 2004), participants described concerns about the capacity of lay people to understand and cope with information about events encountered during police work, which constrained talking. Indeed, some noted that friends or family members did “not want to know” about events at work because it made them feel uncomfortable or worry about the other's safety.

Supportive interactions with others were those where the person listened non-judgementally and offered empathic, validating responses; such qualities have been identified across the range of good informal and formal helping relationships (Barker & Pistrang, 2002). However, in many cases officers chose not to talk to others in detail, yet the perception that they *could* was what seemed crucial to feeling supported. This is consistent with previous research indicating the importance of “perceived” (availability), rather than “received” (actual), social support in relation to PTSD (Prati & Pietrantoni, 2010), although it is not readily explained by the social–cognitive processing model (Lepore, 2001) which emphasises actual talking as the vehicle for emotional processing.

### Limitations and future research

Caution must be exercised in generalising the findings of this study, which recruited a small number of police officers from two conurbations in the UK. The higher ratio of women officers than the national average (Dhani & Kaiza, 2011), and the use of a snowballing approach (entailing a common connection between participants) may have influenced the opinions expressed. Officers appeared to talk candidly about their experiences, but it is impossible to eradicate the influence of the researcher and participant characteristics (e.g., social desirability) on the data collected.

The study focused on officers without a history of PTSD, which was ascertained by verbal report. Future research would benefit from including a more reliable, quantitative measure of PTSD symptoms and examining whether the experiences of supportive/unsupportive interactions differ for those with and without a diagnosis of PTSD. Poor social support may be both a cause and a consequence of PTSD (Kaniasty & Norris, 2008), and therefore longitudinal research is needed.

Future research would also benefit from a mixed-methods approach, incorporating both qualitative and quantitative measures of social support. A measure of social constraints (Lepore & Revenson, 2007) could be used alongside qualitative interviews to obtain a systematic picture of obstacles to support. Observational methods, e.g., recording interactions, would also be useful to address the limitations of retrospective recall of complex social interactions (Pistrang & Barker, 2005).

## Conclusions

The findings of this study provide some tentative insights into the processes of social support that may contribute to the resilience of police officers following traumatic incidents, as well as constraints to seeking and receiving adequate support. Incidents that caused most distress were those with personal relevance, indicating a need to empower officers to seek support at these times and highlighting the limitations of formal support structures being employed in a blanket fashion after “major” incidents. Given that supervisors are a key resource in promoting individual and team welfare, training and support for supervisors to detect distress and foster supportive workplace environments is essential. Although the accounts in the present study suggest that humour and indirect talk help to mitigate the emotional impact of distressing events, police officers are likely to benefit from a social ecology in which emotional expression is more acceptable.
